# Hydrogel supplemented with human platelet lysate enhances multi-lineage differentiation of mesenchymal stem cells

**DOI:** 10.1186/s12951-022-01387-9

**Published:** 2022-04-02

**Authors:** Tong Lei, Yanyan Liu, Shiwen Deng, Zhuangzhuang Xiao, Yanjie Yang, Xiaoshuang Zhang, Wangyu Bi, Hongwu Du

**Affiliations:** 1 School of Chemistry and Biological Engineering, University of Science and Technology Beijing, 112 Lab, Lihua BLDG, No.30 Xueyuan Road, Haidian District, Beijing, 100083 China; 2 Daxing Research Institute, University of Science and Technology Beijing, Beijing, 100083 China; 3 Kangyanbao Stem Cell (Beijing) Co., Ltd, Beijing, 102629 China

**Keywords:** HPL, SHED, Secretome, Osteogenesis, Angiogenesis, Neural-like cells

## Abstract

Stem cells from human exfoliated deciduous teeth (SHED) can be used as a potential clinical material. But the use of xenogeneic ingredients will increase the risk of zoonotic disease transmission. Human platelet lysate (HPL) is a potential surrogate and used in human cell expansion with reliability in clinical applications. In this study, we synthesized chitosan/gelatin/gellan gum hydrogel supplemented with HPL and investigated the effect of 3D culture for SHED. TMT-tagged proteomics was used to decipher the secretome protein profiles of SHEDs and a total of 3209 proteins were identified, of which 23 were up-regulated and 192 were down-regulated. The results showed that hydrogel supplemented with HPL promoted SHED proliferation. After induction, the hydrogel coating contributed to osteogenic differentiation, adipogenic differentiation and differentiation into neural-like cells of SHED. SHED encapsulated in a hydrogel promotes migration and angiogenesis of HUVEC. In conclusion, our research found that hydrogel supplemented with HPL can be used as a method for SHED in standardized production and can contribute to the clinical application of SHED in cell therapy.

**Supplementary Information:**

The online version contains supplementary material available at 10.1186/s12951-022-01387-9.

## Introduction

Stem cells from human exfoliated deciduous teeth (SHED), a kind of mesenchymal stem cells isolated from teeth, can be used as a potential clinical material due to the excellent characteristics including highly proliferation ability, multi-lineage differentiation potential and immunomodulation [1–3]. SHED is a promising material source for tissue regeneration due to it is involved in osteogenesis and angiogenesis [4]. Fibroblast growth factor-2 (FGF-2) and vascular endothelial growth factor (VEGF) can enhances the vascular differentiation and osteogenic differentiation of SHED. SHED can be induced by angiogenic differentiation through efficient activation of Wnt/β-catenin signaling. Furthermore, activation of Wnt signaling can induce vascular differentiation of postnatal mesenchymal stem cells [5]. In particular, as mesenchymal stem cells develop from neural crest in embryonic period, SHED has shown great potential for application in neurodegenerative disease, such as Alzheimer's disease, Parkinson's disease, Huntington's disease and so on [6, 7].

The primary evaluation factor of stem cell therapy strategy is safety, which leads to the use of heterologous substances in cell culture media that should be seriously considered [8]. Xenogeneic ingredients, especially fetal bovine serum (FBS), are commonly used as growth factor supplements for cell culture. However, FBS will increase the risk of zoonotic disease transmission during the use of animal-derived products, including the risk of immune response, viruses, bacteria and prions [9]. In addition, FBS is susceptible to batch variation, resulting in poor repeatability [10]. Human platelet lysate (HPL) is a good alternative to supplement stem cell culture medium for FBS. First of all, HPL can be easily separated and obtained from apheresis products and buffy coat in the form of pooled blood [10]. Secondly, HPL is composed of abundant growth factors, such as nerve growth factor (NGF), neurotrophin 3 (NTF3), brain derived growth factor (BDNF), and glial derived neurotrophic factor (GDNF) and so on. These abundant growth factors provide a suitable environment to support cell viability, proliferation, anti-aging, axonal elongation, genome and immunophenotypic stability. Furthermore, previous reports found that HPL showed reliability to be applied to stem cell culture supplements in quality control, safety and GMP-compliant HPL production release standards [11–13].

Three-dimensional (3D) culture of stem cells is considered to be a suitable way of cell growth and differentiation that mimics the in vivo environment [14]. In this study, non-covalent interactions, including electrostatic, hydrophobic interactions, and hydrogen bonding, were used to prepare hydrogel [15]. We designed a hydrogel, which were prepared from three biomacromolecules, including gelatin, chitosan, and gellan gum (GG). Gelatin is a molecule derived from the abundant extracellular matrix protein collagen, and gellan gum is able to form hydrogel with tunable mechanical properties. Chitosan can support the maintenance of proliferative capacity and pluripotency of stem cells in long-term culture [16–20]. They are widely used in regenerative medicine engineering. We previously confirmed that proteomics was convenient for interpreting the protein expression profile of tooth-derived stem cells [21]. The purpose of this study is to explore the effects of HPL on the cell proliferation, adipogenic differentiation, osteogenic differentiation, vascular differentiation and neural-like cells differentiation of SHED, and to interpret the impact of Chitosan/gelatin/gellan gum hydrogel supplemented with HPL on the protein profile of SHED through quantitative proteomics. Our study will help to promote the application of 3D culture supplemented with HPL in the standardized production and clinical application of stem cells.

## Materials and methods

### Experimental design and statistical rationale

All studies included three biological replicates. The two experimental groups were: (1) SHED and (2) SHED + HPL. The SHED group was a hydrogel supplemented with components FBS. The SHED + HPL group was the hydrogel supplemented with HPL. Both FBS (Cat. No. C04001-050) and HPL (Cat. No. PLTGOLD100R) were purchased from Biological Industries (BI, Israel). Significance analysis was performed. Differentially expressed proteins, p < 0.05 while fold change ≥ 1.5, were considered significant.

### Hydrogel preparation and stem cell culture

**Fig. 1:**
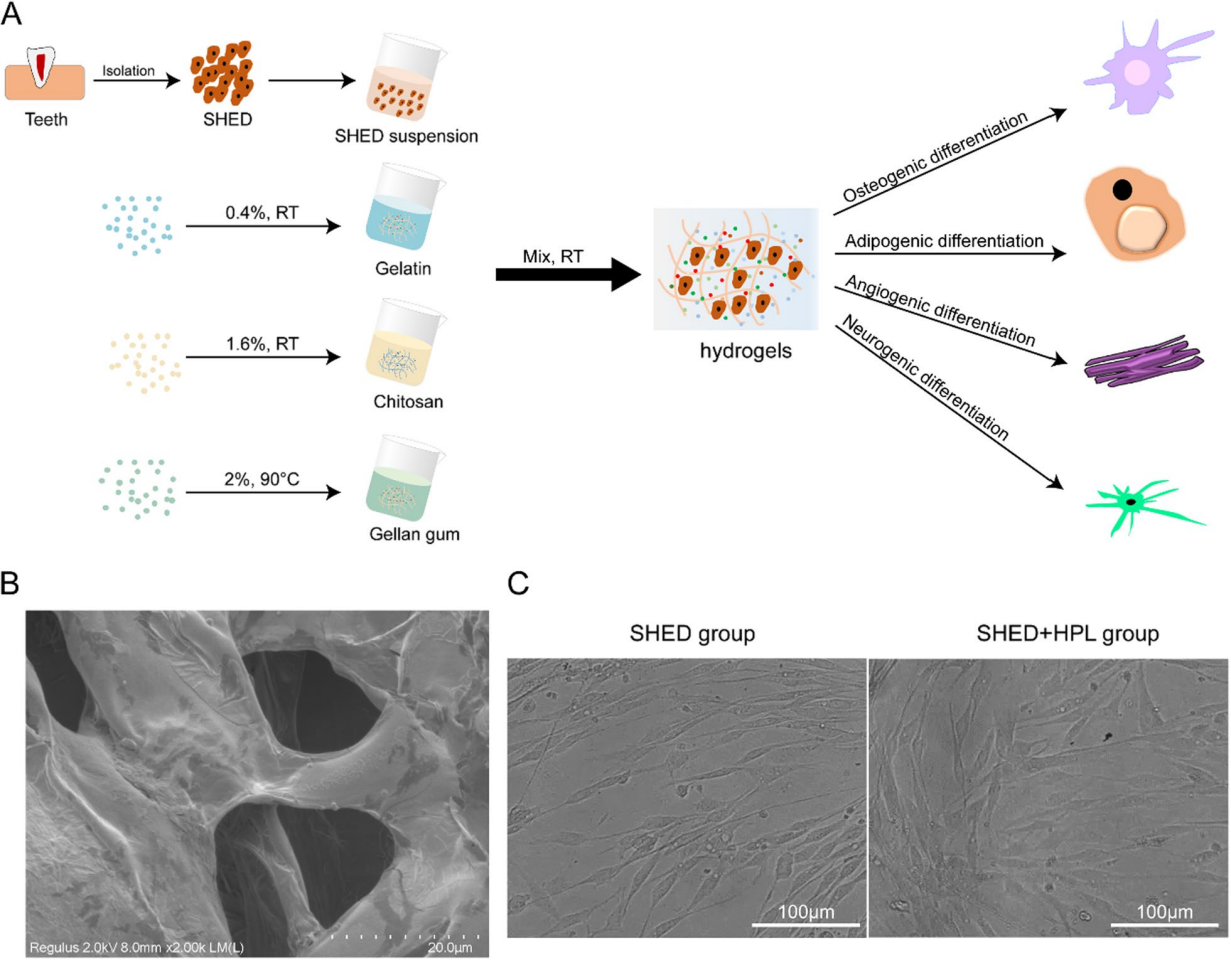
Preparation and characterization of hydrogel. **A** Schematic diagram of preparation of hydrogel and 3D culture and differentiation of stem cells; **B** Scanning electron microscopy imaging of hydrogel. Bar: 20 μm; **C** The appearance of the SHED on the hydrogel with FBS or HPL. Bar: 100 μm. SHED group: SHED cultured on hydrogel supplemented with FBS. SHED + HPL group: SHED cultured on hydrogel supplemented with HPL

Modified gelatin and oxidized gellan gum were prepared as previously reported [15, 22]. A schematic diagram of the preparation of hydrogel and SHED cultures was shown in Fig. 1. Chitosan (Sigma-Aldrich, MO, USA), gelatin (Sigma-Aldrich, MO, USA) and GG (Sigma-Aldrich, MO, USA) solutions were prepared by dissolving each polymer in dulbecco's minimum essential medium (DMEM, BI, Israel) containing 10% FBS or HPL, respectively. Before preparing the hydrogel, the chitosan solution and gelatin polymer solution were filtered at 37 °C using Whatman FP 30/0.2 CA-S sterile filters (Thermo Fisher Scientific, MA, USA), and the GG solution was filtered using Sterivex- GP 0.22 μm filtered Millipore Express sterile filter (Merck Millipore, MA, USA) at 60 °C. Keep the solution at 37 °C, then mix an equal volume (1:1) of the solution by pipette for a few seconds. In 3D culture experiments, cell suspensions were simultaneously mixed with chitosan, gelatin, and GG during gelation. The cell culture medium for all groups was DMEM, and the SHED group was supplemented with 10% FBS, while the SHED + HPL group was supplemented with 5% HPL. After approximately 20 min of gelation time, spread cell culture medium over the samples.

### Scanning electron microscopy

After vacuum freeze-drying, the samples were adhered to the conductive tape of the scanning electron microscope (SEM) base, and were coated by ion sputtering instrument and observed by scanning electron microscope. A scanning electron microscope (SEM, IdC-8010, Japan) was used to observe the physical morphology of the hydrogel at an acceleration voltage of 3 kV.

### Cell isolation and culture

The stem cells from human exfoliated deciduous teeth were separated according to the previous method [1]. This study was approved by the ethical committee of Peking University Third Hospital, and all the participants was obtained informed consent (2021144-02). The study was conducted in accordance with ethical approval granted by the ethical committee of Peking University Third Hospital and followed the Declaration of Helsinki and informed consent was taken from all individual participants. In short, the healthy deciduous teeth from children between the ages of 6 and 8 with informed consent were collected and preserved. The pulp was exposed after the crown was opened, and then the pulp tissue was extracted and cut into pieces. The digestive mixture, containing 0.3% collagenase I and 0.4% dispase (Sigma-Aldrich, MO, USA), was used to prepare single cell suspension by overnight digestion. SHED was seeded in a 7 cm^2^ petri dish (Eppendorf, USA) and was inoculated in DMEM (BI, Israel) supplemented with 10% FBS, 1% 100 U/mL penicillin and 100 mg/mL streptomycin (BI, Israel), and 100 μmol L-ascorbic acid (BI, Israel). SHED was placed in an incubator with 37 ℃ constant temperature, specific humidity and 5% CO_2_. SHED was expanded and cryopreserved after 3–4 weeks in culture. Passages 3 to 5 of SHED were used in the experiments.

### Cell proliferation test

A total of 1 × 10^4^ cells of SHED were seeded in 96-well plates and covered with DMEM supplemented with 10% FBS or 5% HPL. After 24 h of culture, methylthiazolyldiphenyl-tetrazolium bromide (MTT, Solarbio, China) was added to the culture medium and incubated for 4 h at 37 °C. After discarding the supernatant, Formazan was dissolved in DMSO (Sigma-Aldrich, MO, USA) and the OD value at 490 nm was measured in the microplate reader. Independent 3 repeated experiments were performed.

### Flow cytometry

SHED were harvested using 0.05% Trypsin–EDTA (BI, Israel) and washed twice in PBS (BI, Israel). Cells were filtered through a 70 mm cell strainer. A total of 1 × 10^5^ SHED cells were prepared into a single cell suspension, fixed with 4% paraformaldehyde (Solarbio, China) and washed with PBS three times. The cells were labeled with CD14 (PE, BD Biosciences, USA), CD19 (PE, BD Biosciences, USA), CD34 (PE, BD Biosciences, USA), CD45 (PE, BD Biosciences, USA), CD73 (FITC, BD Biosciences, USA), CD90 (FITC, BD Biosciences, USA), CD105 (FITC, BD Biosciences, USA) and HLA-DR (PE, BD Biosciences, USA). And the intensity of SHED was analyzed by flow cytometry (BD Biosciences, NJ).

### Osteogenesis and adipogenesis induce differentiation

The hydrogel coating was seeded on the bottom of the culture plate. Osteogenic and adipogenic differentiation of SHED were performed according to the human dental pulp stem cell osteogenic differentiation medium kit (Cyagen Biosciences, China) and the human dental pulp stem cell adipogenic differentiation medium kit (Cyagen Biosciences, China), respectively. A total of 1 × 10^6^ cells were seeded in 6-well plates and the induction medium was replaced after the cells grew adherently. Following the protocol for osteogenic differentiation, replace the fresh induced differentiation medium every 3 days. Similarly, according to the adipogenic differentiation protocol, change the fresh induction medium A for 3 days, and then change the fresh induction medium B for 1 day. The calcium nodules and lipid droplets of the cells were stained by Alizarin Red (Cyagen Biosciences, China) and Oil Red O (Cyagen Biosciences, China) after 3–5 weeks of induction, respectively.

### Differentiation to neural-like cells

A total of 1 × 10^5^ SHED cells were seeded in 12-well plates and were encapsulated in hydrogel. The fresh neural-like cells induction medium, supplemented with EGF (10 ng/mL, Sigma-Aldrich, MO, USA) and bFGF (10 ng/mL, Sigma-Aldrich, MO, USA), was replaced until the cell fusion rate reaches 80–90%. The neural-like cells markers were detected after 2 weeks.

### Peptide preparation and labeling

After SHED were encapsulated in hydrogel and cultured for 3 days, the supernatants were collected and concentrated to extract proteins. A total of 300 μg protein was extracted. Then, the protein solution was cleaved into peptides in trypsin (Sigma-Aldrich, MO, USA) at 37 ℃ overnight after alkylation and methylation by DL-Dithiothreitol (DTT, Macklin, China) and Iodoacetamide (IAA, Macklin, China). Desalting of the peptide solution was performed at Sep-Pak C18 1 cc Vac Cartridge (Waters, USA) and labeled by TMTsixplex™ isobaric label reagent set (Thermo Fisher Scientific, USA) and terminated in hydroxylamine solution (Thermo Fisher Scientific, USA) at room temperature.

### LC–MS/MS and bioinformatics analysis

The peptide was analyzed and identified by LC–MS/MS. After re-dissolved in 1% formic acid (FA, Rhawn, China), the peptide was redistributed in the C18-reversed phase trap Gemini column (Phenomenex, Torrance, CA) and Orbitrap Fusion MS (Thermo Fisher Scientific) fitted with an online Easy-nLC 1000 system (Thermo Fisher Scientific). The raw file was analyzed in Maxquant (version 1.6.2.0) for TMT6-126, TMT6-127, TMT6-128, TMT6-129, TMT6-130 and TMT6-131 based on the human FASTA database and the false discovery rate (FDR) was limited to 0.01. Gene ontology (GO) enrichment and Kyoto Encyclopedia of Genes and Genomes (KEGG) pathway analysis were performed in Database for Annotation, Visualization and Integrated Discovery (DAVID) online tool (https://david.ncifcrf.gov). The protein–protein interaction network was constructed in STRING (https://string-db.org/) and visualized in Cytoscape (version 3.7.2). Weighted correlation network analysis (WGCNA) was performed according to the following procedure. Data files containing protein expression datasets and stem cell characteristics were prepared and organized in a standard format. First, protein abundances were clustered in R software (https://www.r-project.org/) and R studio (https://www.rstudio.com/) to construct weighted gene networks. Second, the correlations and correlation coefficients of protein profiles and groupings were calculated, and important modules were identified and associated with stem cell characteristics.

### Cell migration assay

The hydrogel was spread evenly in the upper insert (Eppendorf, USA). The human umbilical vein endothelial cells (HUVEC) were obtained from the American Type Culture Collection (ATCC, USA) and cultured in DMEM medium. A total of 1 × 10^5^ cells of HUVEC were seeded in a 6-well plate and co-cultured with SHED. The 200μL yellow pipette tip was used to scratch the bottom. Then, SHED was seeded in the insert and transferred to a 6-well plate to form a co-culture system with HUVEC. After 12 h of culture, the migration of cells was observed and the migration rate was calculated. Independent 3 repeated experiments were performed.

### The tube formation assay in vitro

A total 1 × 10^5^ cells of HUVEC was seeded on a 6-well plate pre-covered with SHED-encapsulated hydrogel supplemented with HPL. A co-cultivation system between SHED and HUVEC was constructed. After culturing for 12 h, the angiogenic differentiation of the HUVEC was observed and photographed by a fluorescence inverted phase contrast microscopy (CNOPTEC, China). Independent 3 repeated experiments were performed.

### RNA isolation, reverse transcription and real-time quantitative PCR

Trizol (CWBio, China) was added to lyse the cells for 10 min. Chloroform (Tgreag, China) and isopropanol (Tgreag, China) were used for RNA isolation and precipitation, respectively. The cDNA library was constructed by reverse transcription in a RT-PCR kit (CWBio, China). UltraSYBR Mixture (CWBio, China) was used for fluorescence quantification and signal feedback to detect gene amplification. The relative expression calculation method of mRNA and primer sequence refers to our previous method [23]. The experiment was repeated three times.

### Immunohistochemistry

The cells were washed with PBS and fixed with 4% paraformaldehyde (Solarbio, China). The cells were blocked with 5% goat serum and incubated with primary antibodies for 1 h including GFAP (Beyotime, China) and Nestin (Beyotime, China). The FITC-linked secondary antibody (Beyotime, China) was used to bind to neural markers and the cells were observed on an inverted fluorescence microscope (Evos D840, CNOPTEC, China).

### Statistical analysis

Visualization and analysis of data were performed in Prism software (version 8.0, GraphPad, San Diego, CA, USA). Student’s t test or one-way ANOVA was used to analyze the significance of the data. Values of p < 0.05 were set statistically significant, and p < 0.05*, p < 0.01**, p < 0.001***.

## Results

### Preparation and characterization of hydrogel

We synthesized hydrogel in chitosan, gelatin and gellan gum by non-covalent interaction. Figure 1A shows a schematic diagram of the synthesis of the hydrogel. Tooth-derived SHED was extracted and encapsulated in HPL-supplemented hydrogel and used to study the osteogenic, adipogenic, angiogenic, and neural-like cells differentiation of stem cells under different induction conditions. To understand the microstructure and function of hydrogel, SEM was used to resolve the structure of freeze-dried hydrogel. The results showed that in the dry state, the hydrogel was loose and porous, and the surface was flat and smooth (Fig. 1B). We observed that SHED cultures exhibited fusiform fibroblast-like morphological features on hydrogel supplemented with FBS or HPL (Fig. 1C). These results suggested that our synthesized chitosan/gelatin/GG hydrogel provided a comfortable structure and surface for SHED to survive.

### Secretomic profiles of HPL-supplemented hydrogel for SHED

**Fig. 2:**
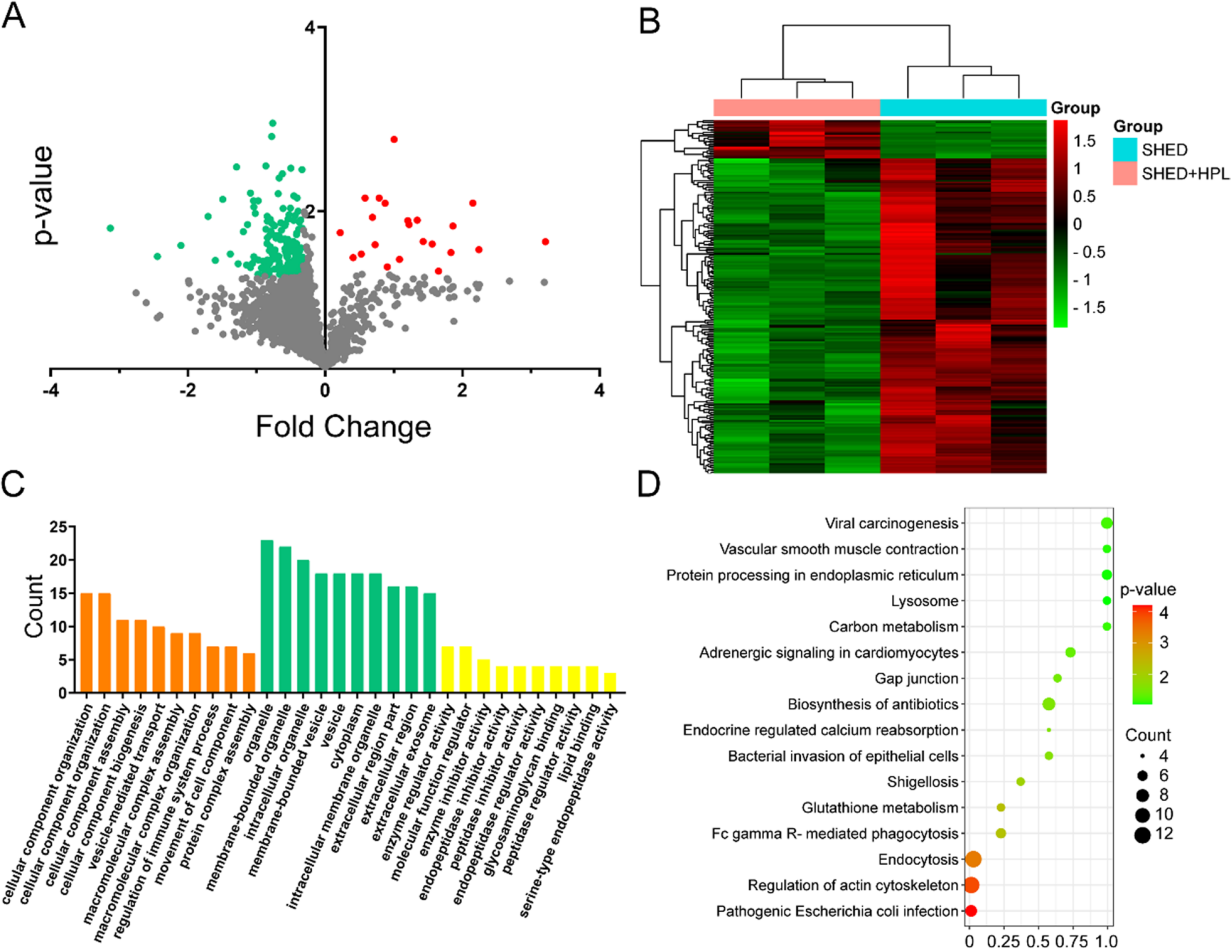
Bioinformatics analysis of protein profile in SHED secretion cultivated at HPL-supplemented hydrogel. **A** The protein expression volcano map of SHED secretion cultivated at HPL-supplemented hydrogel. The abscissa represented the Fold change (logarithm of 2) while the ordinate represented the p-value (negative logarithm of 10). The red balls represented up-regulated DAPs, and the green balls represented down-regulated DAPs in the HPL group compared to the SHED group; **B** Heat map of up-regulation and down-regulation of DAPs; **C** GO enrichment analysis of DAPs, including Biological Process, Cellular Component and Molecular Function; **D** There were a total of 16 pathways in the KEGG analysis of DAPs

**Fig. 3:**
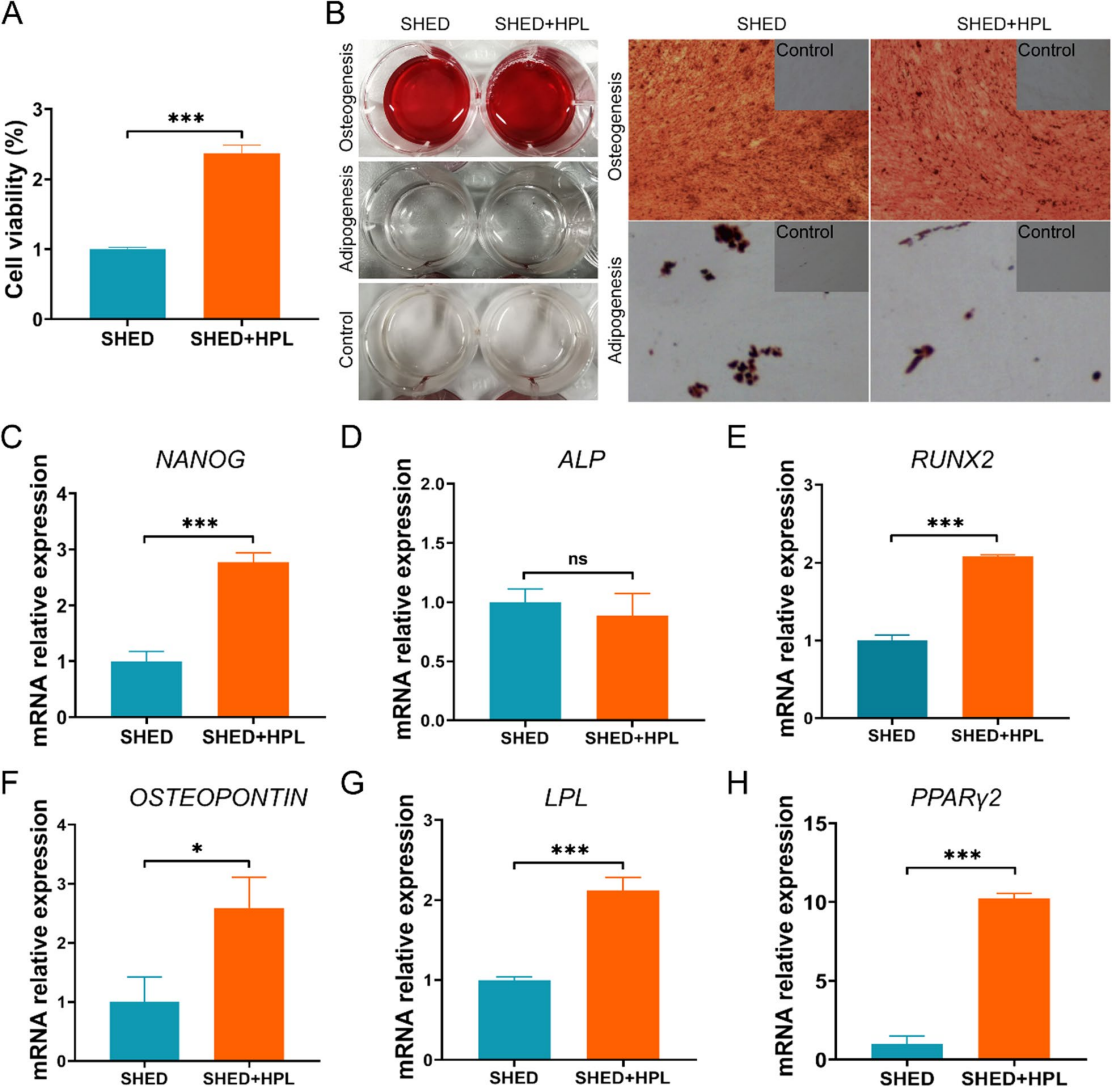
The effects of HPL-supplemented hydrogel on the characteristics of SHED include proliferation, stemness, osteogenic and adipogenic differentiation. **A** Cell proliferation of SHED were performed after cultured on HPL-supplemented hydrogel for 24 h. The ordinate represents the relative cell viability; **B** Alizarin Red and Oil Red O were used for staining to characterize the osteogenic differentiation and adipogenic differentiation of SHED cultured on HPL-supplemented hydrogel. Magnification: 4 × 10; **C** Expression of the mRNA of the cell stemness gene NANOG; **D** The mRNA expression of osteogenic differentiation markers ALP of stem cells; **E** qPCR was used to detect the expression of osteogenic differentiation marker genes RUNX2; **F** The mRNA expression of osteogenic differentiation markers OSTEOPONTIN of stem cells; **G** qPCR was used to detect the expression of adipogenic differentiation genes LPL; **H** The expression of adipogenic differentiation marker genes PPARγ2 was detected by RT-qPCR; *ALP* alkaline phosphatase, *RUNX2* runt-related transcription factor 2, *PPARγ* peroxisome proliferator activated receptor gamma, *LPL* lipoprotein lipase. The data was shown in mean ± SEM. ***p < 0.001, **p < 0.01, *p < 0.05, ns: no significant difference. Three independent experiment repetitions were performed

To understand the molecular expression profile of SHED secretion cultivated at HPL-supplemented hydrogel, we identified and analyzed the protein by mass spectrometry-based quantitative proteomics. A total of 3209 proteins were identified (Additional file 1: Table S1), of which 23 were up-regulated differential abundant proteins (DAPs, Additional file 2: Table S2) and 192 were down-regulated (Fig. 2A, Additional file 3: Table S3). The expression heatmap was shown in Fig. 2B. To understand the information of differential proteins, we performed GO and KEGG analysis. Cell component analysis found that DAPs were mainly located in organelle, membrane-bounded organelle, intracellular organelle, membrane-bounded vesicle, vesicle, cytoplasm, intracellular membrane organelle, extracellular region part, extracellular region and extracellular exosome; Molecular Function found that DAPs were mainly involved in a variety of molecular activities including enzyme regulator activity, molecular function regulator, enzyme inhibitor activity, endopeptidase inhibitor activity, peptidase inhibitor activity, endopeptidase regulator activity, glycosaminoglycan binding, peptidase regulator activity, lipid binding and serine-type endopeptidase activity; DAPs participated in a variety of biological process including cellular component organization, cellular component assembly, cellular component biogenesis, vesicle-mediated transport, macromolecular complex assembly, macromolecular complex organization, regulation of immune system process, movement of cell component and protein complex assembly (Fig. 2C). A total of 16 pathways were found in the KEGG analysis, including Pathogenic Escherichia coli infection, Regulation of actin cytoskeleton, Endocytosis, Glutathione metabolism, Fc gamma R-mediated phagocytosis, Shigellosis, Bacterial invasion of epithelial cells, Endocrine regulated calcium reabsorption, Biosynthesis of antibiotics, Gap junction, Adrenergic signaling in cardiomyocytes, Viral carcinogenesis, Carbon metabolism, Vascular smooth muscle contraction, Protein processing in endoplasmic reticulum and Lysosome (Fig. 2D). We noticed that HPL-supplemented hydrogel promoted SHED growth, proliferation, and secretion of migration-related factors such as platelet factor 4, CD44, and elastin microfibril interfacer 1 (Fig. 3). So, we explored stem cell migration, osteogenic differentiation, adipogenic differentiation, angiogenesis, and neural-like cells differentiation in hydrogel.

### HPL-supplemented hydrogel enhances the proliferation and differentiation of SHED

To explore the impact of HPL-supplemented hydrogel on the characteristics of SHED, we conducted stem cell proliferation experiments, osteogenic and adipogenic differentiation analysis. The results showed that HPL-supplemented hydrogel enhanced cell proliferation of SHED (Fig. 3A). In addition, the Alizarin Red staining results demonstrated that there were observable calcium nodules, which indicated that cultivated at hydrogel can maintain the osteogenic differentiation capacity of SHED after 3–4 weeks of induction (Fig. 3B). In addition, after 4–5 weeks of induction, Oil Red O staining results demonstrated that cultivated at hydrogel can maintain the adipogenic differentiation capacity of SHED (Fig. 3B) RT-qPCR was used to detect gene expression, and the results showed that the expression of stemness gene NANOG (p < 0.05) was up-regulated after culture in HPL-supplemented hydrogel for 3 days (Fig. 3C). Moreover, osteogenesis related genes were significantly up-regulated on HPL-supplemented hydrogel (Fig. 3D–H), including *RUNX2* (p < 0.001) and *OSTEOPONTIN* (p < 0.05), and adipogenic differentiation genes were up-regulated, including *LPL* (p < 0.001) and *PPARγ2* (p < 0.001). In short, HPL-supplemented hydrogel can improve the cell proliferation and stemness in 3D culture. SHED cultured in HPL-supplemented hydrogel enhanced osteogenic and adipogenic differentiation ability after 3–4 weeks of overlaying in differentiation-inducing medium. These results support that hydrogel can be used as carriers for 3D culture and differentiation induction of stem cells.

### The surface markers of hydrogel supplemented with HPL of SHED

To explore the effect of HPL-supplemented hydrogel treatment on the immunophenotype of SHED, we tested the surface markers of SHED by flow cytometry. The expression level of the surface markers of SHED were CD14 (12.12%), CD19 (3.33%), CD34 (0.02%), CD45 (0.32%), CD73 (99.86%), CD90 (99.94%), CD105 (100.00%), HLA-DR (0.28%), and the surface markers of SHED cultured in HPL-supplemented hydrogel were CD14 (0.84%), CD19 (0.60%), CD34 (0.00%), CD45 (0.10%), CD73 (99.84%), CD90 (99.75%), CD105 (99.80%) and HLA-DR (0.00%) (Additional file 4: Table S4). SHED cultivated at HPL-supplemented hydrogel showed positive expression of mesenchymal stem cell markers and negative expression of hematopoietic stem cell markers. The negative markers of SHED under FBS- or HPL-supplemented hydrogel reached the standard of industrial production (less than 2%), which indicated that the hydrogel supplemented with HPL culture was a potential process in stem cell production.

### HPL-supplemented hydrogel promotes migration and angiogenesis of HUVEC

**Fig. 4:**
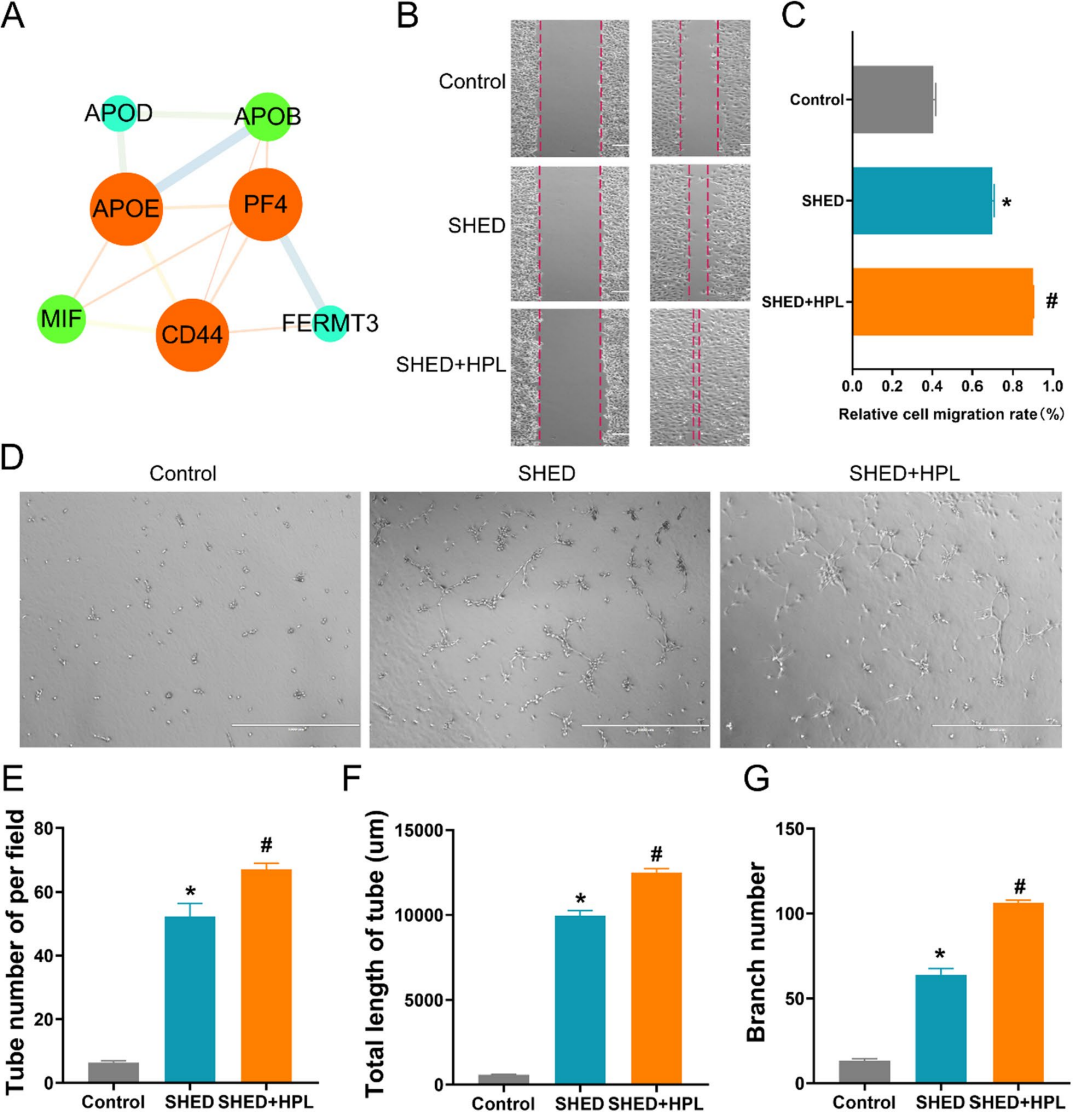
The effect of HPL on the cell migration and angiogenesis of SHED. **A** Proteins related to cell migration and angiogenesis were constructed as a network of interactions, including APOB, APOD, MIF, CD44, PF4, APOE and FERMT3; **B** The cell migration of HUVEC in the co-culture system was observed; **C** The relative cell migration rate was calculated in the co-culture system; **D** The angiogenic differentiation of HUVEC was analyzed in a co-culture system; **E** Angiogenesis were performed statistically to analyze the tube number of per field in vitro; **F** The total length of the tube (μm) was statistically analyzed; **G** The branch number was statistically analyzed; Control group: HUVEC; SHED group: SHED co-culture with HUVEC; SHED + HPL group: SHED cultivated at HPL co-culture with HUVEC; One-way ANOVA was used for statistical significance. The data was shown in mean ± SEM. *: compare to control group, p < 0.05; #: compare to SHED group, p < 0.05

The differentiation potential of stem cells is the basis of tissue regeneration engineering, especially vascular reconstruction. The proteomics results showed that HPL-supplemented hydrogel up-regulated the expression of proteins related to cell migration and angiogenesis in SHED. The results showed that there were widespread interactions between these proteins (Additional file 5: Table S5), including APOB, APOD, MIF, CD44, PF4, APOE and FERMT3 (Fig. 4A). A co-culture system was constructed by SHED and HUVEC to evaluate cell migration and angiogenesis. The statistical results showed that SHED-encapsulated hydrogel supplemented with HPL can improve cell migration rate, the tube number of per field, the total length of the tube, and branch number of angiogenesis in the co-culture system (Fig. 4B–G). The results revealed that SHED can promote the cell migration and angiogenesis of HUVEC (p < 0.05), and HPL-supplemented hydrogel can significantly enhance these benefits (Fig. 4B–C).

### SHED encapsulated in hydrogel significantly enhances differentiation to neural-like cells

**Fig. 5:**
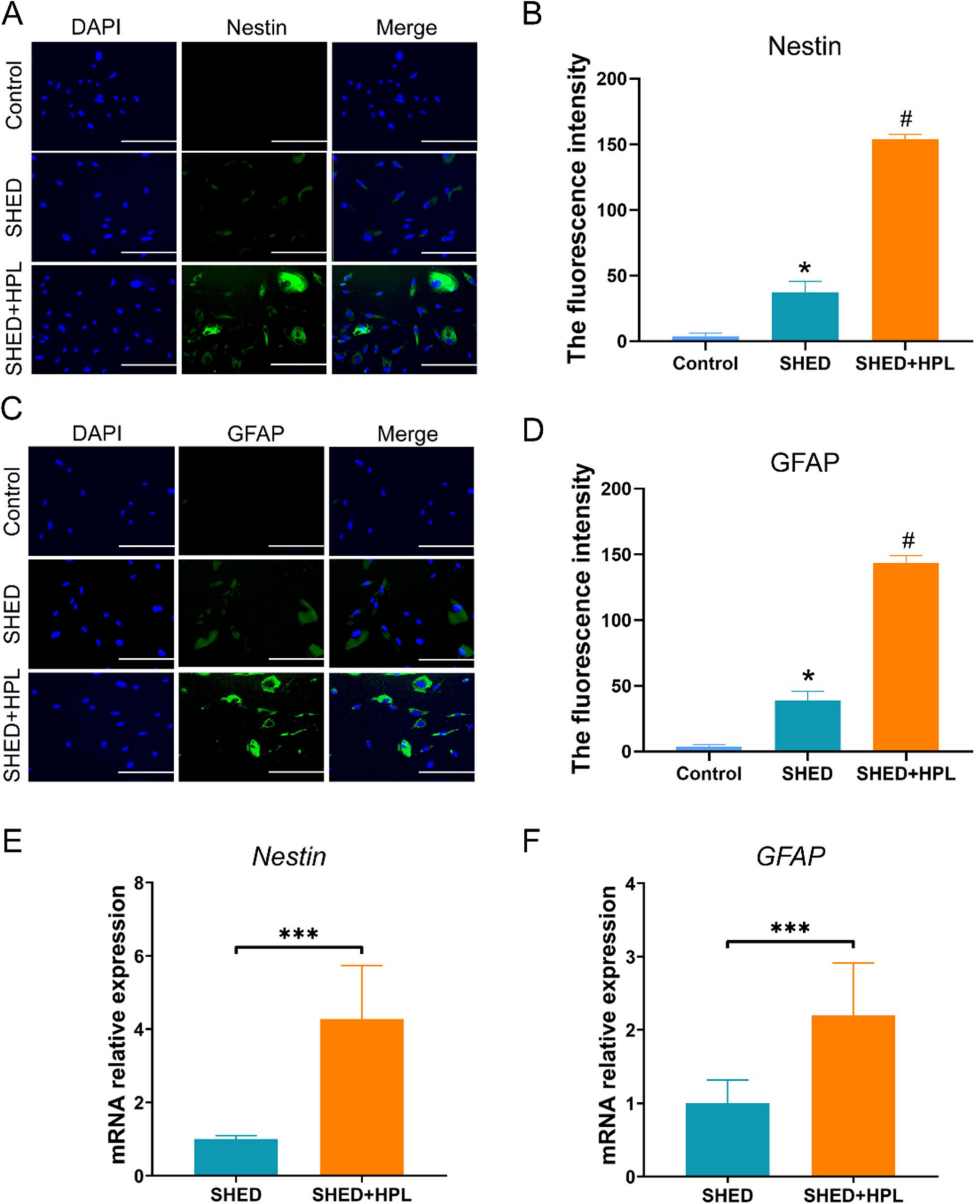
SHED encapsulated in hydrogel was induced to differentiate and detect neuron-related markers. **A** The results of immunofluorescence analysis showed the expression of Nestin markers; **B** Fluorescence intensity statistics of Nestin (FITC). **C** The results of immunofluorescence analysis showed the expression of GFAP. **D** Fluorescence intensity statistics of Nestin (FITC); **E** qPCR was used to detect the gene expression level of *Nestin*; **F** The mRNA level of *GFAP* was analyzed. One-way ANOVA was used for statistical significance. The data was shown in mean ± SEM. ***p < 0.001, **p < 0.01, *p < 0.05. Three independent experiment repetitions were performed

As the mesenchymal stem cells derived from neural crest, the neural-like cells differentiation of SHED is considered to be the basis for the treatment of nerve injury diseases in regenerative medicine. To evaluate the impact of HPL-supplemented hydrogel on the neural-like cells differentiation of SHED, we induced differentiation of SHED by supplementing the EGF and bFGF. After induction for 2 weeks, immunofluorescence results showed that SHED cultured in hydrogel significantly expressed markers Nestin (p < 0.05) and GFAP (p < 0.05, Fig. 5A–D). The qPCR results revealed that hydrogel promoted the mRNA expression levels of *Nestin* (p < 0.001) and *GFAP* (p < 0.001) (Fig. 5D–F).

### Correlation between cell characteristics and protein expression

**Fig. 6:**
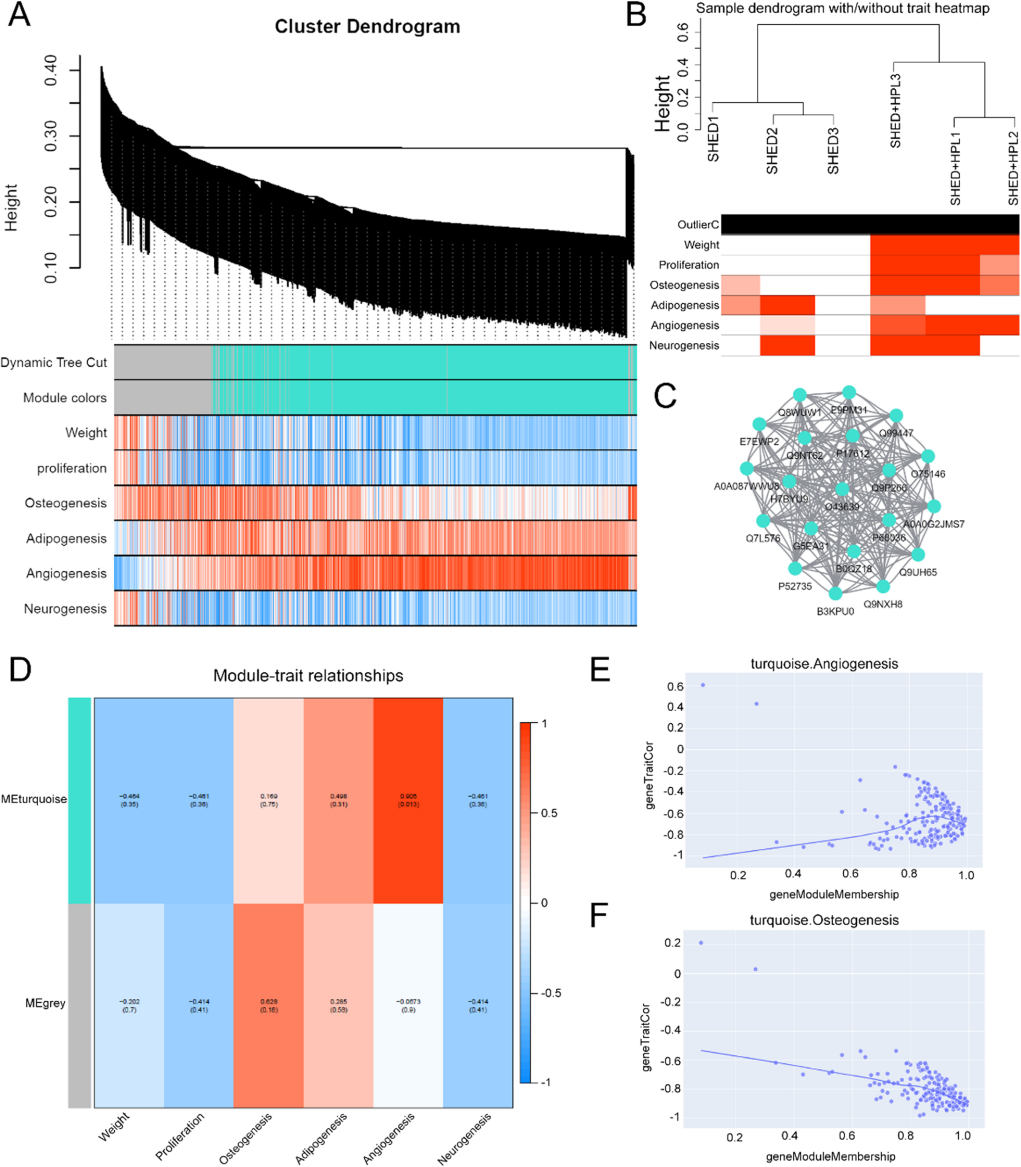
WGCNA was used to analyze the relationship between protein expression in proteomics and the characteristics of SHED. **A** Hierarchical clustering tree bases on the topological overlap dissimilarity. The tree branches of the hierarchical clustering were colored by module membership; **B** The sample dendrogram with/without trait heatmap was shown including SHED group and HPL group; **C** The hub gene module was used to analyze the key 20 proteins interaction network; **D** Relationships between gene modules and cell proliferation, osteogenesis, adipogenesis, angiogenesis or neurogenesis. **E** Heat map trends of key protein expression were shown in turquoise Angiogenesis module; **F** Heat map of key protein expression were shown in turquoise Osteogenesis module

To construct the correlation between cell phenotype and protein profile, we performed WGCNA analysis. Cell proliferation, osteogenesis, adipogenesis, angiogenesis or neurogenesis were used to explore the correlation of protein expression. The results showed that protein abundance was enriched to form a dendrogram and differentiated into 2 modules, including turquoise and grey (Fig. 6A–B). An interaction network was constructed to extract key molecules, including SEC24C, C20orf27, TPM3, HIP1R, SWAP70, TRIO, UBE2L3, BRK1, ATG3, KIAA1462, CPNE1, PCYT2, PDLIM2, CD81, TOR4A, VAV2, CYFIP1, NCK2, SCRIB, and PRKACA (Fig. 6C). The turquoise module was found to have a strong correlation with angiogenesis and osteogenesis (Fig. 6D), which shows a linear correlation with a series of protein expression (Fig. 6E–F).

## Discussion

Mesenchymal stem cells are a potential clinical material used in the treatment of major diseases including Alzheimer’s disease, Parkinson’s disease and Vision Loss, bone wound, COVID-19, spinal cord injury [24–28]. Tooth tissue, especially the natural deciduous tooth pulp, is an excellent source to extract and isolate mesenchymal stem cells [1]. Pulp samples can be easily obtained in dental medical waste, and there are no ethical issues. SHED is believed to have stronger proliferation and osteogenesis compared to bone marrow mesenchymal stem cells, and is used in regenerative medicine transplantation strategies including diabetes, wound repair, neural injuries, liver fibrosis, and Cohrn's disease, bone tissue engineering [29–31].

The conventional SHED culture system is constructed by supplementing FBS in the basal medium to meet the nutritional requirements of cell growth and proliferation. Our results showed that HPL can promote cell proliferation, osteogenic differentiation and adipogenic differentiation compared to FBS. Inhibitor supplement experiments found that HPL promotes cell proliferation through phosphorylation of ERK1/2, JNK, p38 and AKT pathway [32]. What's more, Gelatin-methacryloyl (GelMA) hydrogel supplemented with HPL can significantly improve cell migration and proliferation in a 3D culture system [33]. Cell stemness was maintained by protecting mitochondrial function, which may be upregulated by HGF and SCF in HPL by activating PI3K/AKT, ERK1/2 and STAT3 signaling pathways [34]. These results indicated that HPL was a powerful alternative to FBS and was used in the production of SHED.

Bone tissue repair engineering is the most common clinical application of dental stem cells, especially craniofacial bone repair. SHED showed strong bone formation and repair capabilities, which can be enhanced by FGF2 and hypoxia through WNT/β-catenin signaling [5, 35]. Both HPL and outdated platelet concentrates can enhance cell proliferation and osteogenic differentiation [36].

A total of 16 signaling pathways were identified in proteomics in the KEGG analysis of DAPs. We noticed that the down-regulated proteins of DAPs were mainly clustered in some signaling pathways, such as Escherichia coli infection, Shigellosis and Bacterial invasion. The downregulated proteins involved in these pathways included ARPC3, TUBB3, TUBB4A, NCK2, ARHGEF2, MAPK3, ELMO2, CLTB in HPL-treated SHED. Medium supplemented with FBS easily leads to cross-infection and spread of cells [37]. However, the KEGG results showed that these infection-related proteins were down-regulated and decreased in HPL, suggesting that HPL was a reliable alternative to FBS.

Proteomics interpreted the protein expression profile of SHED. Quantitative proteomics showed superiority in the study of dental stem cells [38]. The combined use of proteomics and WGCNA analysis can be used to interpret the relationship between protein expression and stem cell characteristics, including cell proliferation, osteogenic differentiation, adipogenic differentiation, angiogenesis, and neural differentiation [21, 23]. A series of proteins were found to be related to cell differentiation. However, high-throughput screening results require further identification of the relationship between molecules and phenotypes.

Our results supported that HPL-supplemented hydrogel promoted SHED proliferation and differentiation, but these benefits were also demonstrated in other mesenchymal stem cells. Medium supplemented with HPL promoted adipose-derived mesenchymal stem cells (ADSCs) to show significantly higher proliferation rates, showing statistically higher levels of neurotrophic factors BDNF, glial cell-derived growth factor (GDNF), and nerve-derived growth factor (NFG) secretion, compared to FBS-supplemented medium [39]. Cell spheroids encapsulated in HPL-supplemented PEG hydrogel exhibited initial ADSCs growth and ultimately successful colonization. ADSCs cultured on HPL-supplemented PEG hydrogel showed high speed migration, covered long distances, and migrated only in the direction of the HPL-loaded PEG hydrogel [40]. In addition, In the in vitro co-culture system, HPL supplemented medium promoted the angiogenesis of gingival mesenchymal stem cells [41]. HPL hydrogel stimulates pro-angiogenic activity by promoting the growth and invasion of human MSCs in a 3D environment and enhancing endothelial cell sprouting in vitro. The combination of HPL and human MSCs improved local tissue perfusion after 8 days in a mouse model of hindlimb ischemia, which supports the use of HPL hydrogel as scaffolds for MSC delivery to promote angiogenesis [42].

In summary, we synthesized chitosan/gelatin/gellan glue hydrogel supplemented with HPL and investigated the effect of 3D culture on the proliferation, multi-lineage differentiation and secretome of SHED. We found that a total of 3209 proteins were identified, of which 23 were up-regulated and 192 were down-regulated. The results showed that 3D culture promoted SHED proliferation and enhanced the expression of stemness genes *NANOG,* and low expression of negative surface markers in flow cytometry suggested that 3D culture was suitable for standardized production. After 3–4 weeks of induction, the hydrogel coating contributed to osteogenic differentiation of SHED with the expression of osteogenic-related genes *RUNX2* and *OSTEOPONTIN*. In addition, the hydrogel supplemented with HPL enhanced the adipogenic differentiation of SHED with the expression of adipogenic-related genes *LPL* and *PPARγ2*. After the addition of neural-inducing differentiation factors, SHED encapsulated in hydrogel supplemented with HPL differentiated into neural-like cells and expressed neural markers Nestin and GFAP. We established the relationship between protein expression and proliferation, osteogenesis, adipogenesis, angiogenesis or neurogenesis by WGCNA, and found related protein series including EMILIN1, PF4 and CD44. In conclusion, our research found that hydrogel supplemented with HPL can be used as a method for SHED in standardized production and can contribute to the clinical application of SHED in cell therapy.

## Supplementary Information

**Additional file 1**: **Table S1.** Protein profile of SHED cultured at HPL.

**Additional file 2**: **Table S2.** Up-regulated differential abundance proteins in HPL treated SHED.

**Additional file 3**: **Table S3.** Down-regulated differential abundance proteins in HPL treated SHED.

**Additional file 4**: **Table S4.** The expression positive rate of surface markers in flow cytometry.

**Additional file 5**: **Table S5.** Proteins related to cell migration and angiogenesis in term of protein profile.

## Data Availability

The mass spectrometry proteomics data have been deposited to the ProteomeXchange Consortium via the PRIDE partner repository with the dataset identifier PXD026687.

## Abbreviations

SHED: Stem cells from human exfoliated deciduous teeth ()
FBS: Fetal bovine serum
HPL: Human platelet lysate
NGF: Nerve growth factor
NTF3: Neurotrophin 3
BDNF: Brain derived growth factor
GDNF: Glial derived neurotrophic factor
GG: Gellan gum
DMEM: Dulbecco's minimum essential medium
SEM: Scanning electron microscope
FTIR: Fourier transform infrared
ATR: Attenuation total reflection
GO: Gene ontology
KEGG: Kyoto Encyclopedia of Genes and Genomes
DAPs: Differential abundant proteins
WGCNA: Weighted correlation network analysis
HGF: Hepatocyte growth factor,
SCF: Stem cell factor
HUVEC: Human umbilical vein endothelial cells
